# Chemical Addressability of Ultraviolet-Inactivated Viral Nanoparticles (VNPs)

**DOI:** 10.1371/journal.pone.0003315

**Published:** 2008-10-02

**Authors:** Chris Rae, Kristopher J. Koudelka, Giuseppe Destito, Mayra N. Estrada, Maria J. Gonzalez, Marianne Manchester

**Affiliations:** Department of Cell Biology, and Center for Integrative Molecular Biosciences, The Scripps Research Institute, La Jolla, California, United States of America; University of Minnesota, United States of America

## Abstract

**Background:**

Cowpea Mosaic Virus (CPMV) is increasingly being used as a nanoparticle platform for multivalent display of molecules via chemical bioconjugation to the capsid surface. A growing variety of applications have employed the CPMV multivalent display technology including nanoblock chemistry, *in vivo* imaging, and materials science. CPMV nanoparticles can be inexpensively produced from experimentally infected cowpea plants at high yields and are extremely stable. Although CPMV has not been shown to replicate in mammalian cells, uptake in mammalian cells does occur *in vitro* and *in vivo*. Thus, inactivation of the virus RNA genome is important for biosafety considerations, however the surface characteristics and chemical reactivity of the particles must be maintained in order to preserve chemical and structural functionality.

**Methodology/Principal Findings:**

Short wave (254 nm) UV irradiation was used to crosslink the RNA genome within intact particles. Lower doses of UV previously reported to inactivate CPMV infectivity inhibited symptoms on inoculated leaves but did not prohibit systemic virus spread in plants, whereas higher doses caused aggregation of the particles and an increase in chemical reactivity further indicating broken particles. Intermediate doses of 2.0–2.5 J/cm^2^ were shown to maintain particle structure and chemical reactivity, and cellular binding properties were similar to CPMV-WT.

**Conclusions:**

These studies demonstrate that it is possible to inactivate CPMV infectivity while maintaining particle structure and function, thus paving the way for further development of CPMV nanoparticles for *in vivo* applications.

## Introduction

Virus particles have received increasing attention as natural platforms for molecular display in an extensive variety of nanotechnology applications (reviewed in [1]). Cowpea mosaic virus (CPMV), a plant virus, has been developed as a programmable nanoparticle platform for vaccine development [2]–[7], and immunoassay detection [8]. Furthermore, we recently demonstrated CPMV bioavailability and systemic tissue trafficking in mouse [9] as well as the use of CPMV as a biosensor agent for *in vivo* vascular and tumor imaging and tumor targeting [10], [11].

CPMV is a comovirus in the picornavirus superfamily, and infects the cowpea plant *Vigna unguiculata*. The crystal structure of the CPMV capsid reveals icosahedral symmetry and a 31 nm diameter particle consisting of 60 copies each of a large (L) and a small (S) capsid protein [12]. CPMV is stable in a variety of environmental conditions such as low pH, temperatures up to 60°C, and organic solvents such as dimethyl sulfoxide [13]. Infection of cowpea plants typically yields 1 mg of CPMV (approximately 10^14^ virus particles) per gram of infected leaf tissue [7].

The CPMV capsid may be manipulated by modification of cDNAs encoding the viral genomic RNAs. The positive-sense bipartite single strand RNA genome consists of RNA-1 (5.9 kb), which codes for several non-structural proteins including the viral protease and polymerase, and RNA-2 (3.6 kb), which codes for the viral L and S capsid proteins and the movement protein [14]. Several sites in the L and S proteins have been shown to be amenable to the insertion of heterologous sequences by genetic manipulation of cDNA clones of RNA-2 [7] and have facilitated the development of CPMV for a variety of applications such as vaccines [2]–[4], [6], [15]–[17], nanoscale chemical building blocks [13], [18]–[22], and inorganic-organic nanoparticle hybrids [23], [24]. Furthermore, each viral capsid contains 300 addressable lysine residues [25], which allows for efficient and stable isothiocyanate and NHS-ester coupling reactions to the amine residue of exposed lysines. Such reactions are used frequently in ligand conjugation including fluorescent dyes [9], [10], [26] and click chemistry [27], [28]. A recent study also employed reactive surface carboxylates on CPMV for bioconjugation [29].

The use of plant viruses for nanobiotechnology also includes viruses such as cowpea chlorotic mottle virus (CCMV), potato virus X, tobacco mosaic virus (TMV), and brome mosaic virus (BMV). Initial studies have been primarily for vaccine applications, however TMV, CCMV and BMV in particular have been increasingly used as chemical scaffolds for *in vitro* and *in vivo* use as well (reviewed in [1], [30]). Thus a growing spectrum of virus capsids with a diversity of size, shape, and chemical character are becoming available for material science and nanotechnology.

The use of CPMV for biological applications requires inactivation of infectivity. CPMV cannot be assembled *in vitro*, nor can virus-like particles be efficiently produced in heterologous systems. Empty particles, also known as top component based on their migration in sucrose gradients, may be recovered or artificially generated by high-pH treatment [31]. The pH method may also be used to reduce infectivity [32], however high pH-treated particles are susceptible to changes in capsid morphology, and like most virus-like particles that are devoid of RNA they are relatively less stable substrates for chemistry [31]. Methods typically employed for inactivation of virus or bacteriophage infectivity include chemical treatment (formalin, beta propiolactone, chlorine, hydrogen peroxide, ammonia, and sodium hypochlorite), exposure to extremes of pH or heat, desiccation, proteolytic degradation, isotope irradiation or UV irradiation [33]. UV radiation is absorbed by nucleic acids and induces dimerization of adjacent pyrimidines by cyclobutyl ring formation or other photoproduct linkages, which damage the nucleic acid strand and thus inhibit transcription [34]. RNA-protein crosslinking may also occur.

A previous study indicated that a CPMV-based vaccine that displayed an epitope from canine parvovirus and had been inactivated by UV could still induce protective antibody responses [5]. However, that study did not examine whether the chemical reactivity characteristics of the exterior or interior capsid surfaces of UV-treated CPMV were maintained. Such reactivity has been shown to be favored and required for a variety of nanobiotechnology applications for viral nanoparticles *in vivo* [26], [35]–[37]. The aim of the present study was to inactivate viral infectivity without disturbing the structural integrity or the reactivity of the capsid surface, specifically the exposed and chemically addressable surface residues. UV light (254 nm) was used to crosslink the viral RNA, and the dose-dependence of infectivity was measured by infecting *Vigna unguiculata* seedlings. The intactness and chemical functionality of the inactivated virions was then assessed by size-exclusion chromatography, transmission electron microscopy, and the surface reactivity was quantified using *N*-hydroxysuccinimidyl ester conjugation of a fluorophore.

## Results

### CPMV particles exhibit diminished infectivity following UV irradiation

**Figure 1:**
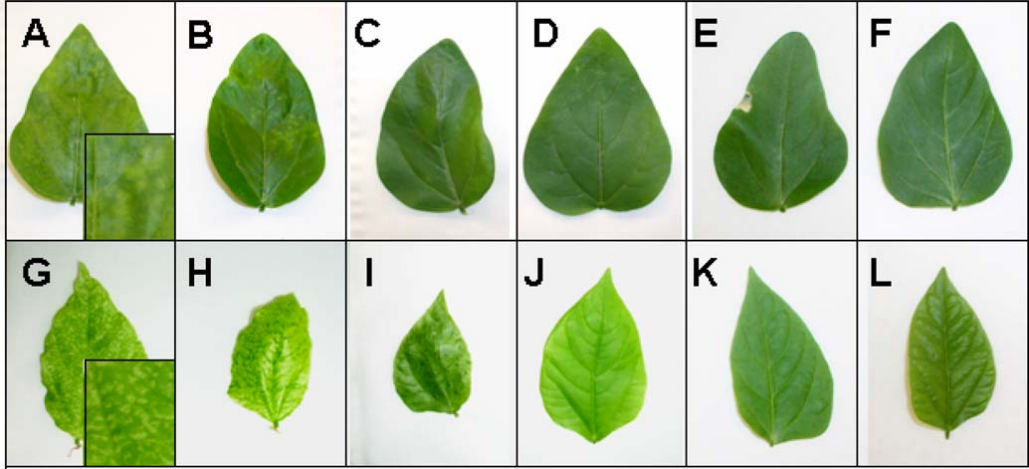
UV-inactivation of CPMV infectivity. Primary leaves (panels A–F) were inoculated with CPMV and the presence of symptoms on these and secondary leaves (panels G–L) of the plant were monitored. Leaves were inoculated with CPMV that had been treated with the following doses of UV irradiation: 0 J/cm^2^ (positive control; panels A, G), 0.72 J/cm^2^ (panels B, H), 1.0 J/cm^2^ (panels C, I), 2.0 J/cm^2^ (panels D, J), 2.5 J/cm^2^ (panels E, K) or no infection (panels F and L). Insets in panels A and G show representative symptoms at approximately 3× magnification. Results shown are representative of five independent experiments with similar results.

CPMV particles are not sensitive to many standard methods of virus inactivation (e.g. hypochlorite or peptidase treatment [9], and methods that preserve capsid integrity such as formaldehyde treatment inhibit subsequent chemical bioconjugation. To determine the dose-response of inactivation by short-wave UV irradiation, samples of purified CPMV diluted to 2 mg/ml concentration were irradiated at various doses of 254 nm UV light as follows: 0 J/cm^2^ (control), 0.06 J/cm^2^, 0.12 J/cm^2^, 0.18 J/cm^2^, 0.36 J/cm^2^, 0.72 J/cm^2^, 1.0 J/cm^2^, 2.0 J/cm^2^, and 2.5 J/cm^2^, (Table 1). Following irradiation, virus samples were directly inoculated into bruised primary leaves of 9 day old cowpea plants and were monitored daily thereafter for the appearance of symptoms on inoculated primary and secondary leaves, as well as reduced growth in secondary leaves indicating systemic spread of CPMV. No reduction in symptoms compared to control was observed for those plants that were inoculated with CPMV receiving doses of 0.06 J/cm^2^, 0.12 J/cm^2^ and 0.18 J/cm^2^ of UV irradiation (Table 1). A decrease in the amount of lesions in primary leaves was observed in virus samples that received 0.36 J/cm^2^ and 0.72 J/cm^2^ UV irradiation, but both showed only a minimal reduction in the number of lesions in secondary leaves (Figure 1, Table 1). CPMV samples that received 1.0 J/cm^2^, 2.0 J/cm^2^, and 2.5 J/cm^2^ doses showed no lesions in inoculated leaves (Figure 1, Table 1). However, the sample receiving 1.0 J/cm^2^ produced a small amount of lesions and a decrease in size of secondary leaves (Figure 1I) whereas the sample receiving 2.0 J/cm^2^ or higher showed no signs of infection in any leaves with the plants displaying no symptoms (Figure 1D, 1J, Table 1).

**Table 1:** Symptoms in inoculated primary leaves and secondary leaves following UV irradiation of CPMV.

UV Dose (J/cm^2^)	Symptoms in 1° leaves	Symptoms in 2° leaves
0 (WT control)	+^a^	+
0.06	+	+
0.12	+	+
0.18	+	+
0.36	+/−	+
0.76	+/−	+
1.0	−	+/−
2.0	−	−
2.5	−	−

a for all doses, +: more than 5 symptomatic lesions per square cm; +/−: less than 5 lesions per square cm; −: no lesions.

Additional experiments performed using a local lesion host for CPMV, *Phaseolis vulgaris var. pinto* (pinto bean), a host that permits CPMV replication in inoculated primary leaves but does not permit systemic spread of virus, showed similar results (Supporting information files Text S1 and Figure S1). Thus samples treated with 2.0 J/cm^2^, and 2.5 J/cm^2^ doses of UV irradiation were further investigated for their surface chemical reactivity and are denoted as CPMV-UV_2.0_ and CPMV-UV_2.5_ respectively for the remainder of the study. Untreated CPMV is denoted CPMV-WT.

Given that it would be appealing to use virus-based therapeutics and vaccines in an edible form, and also that we previously demonstrated that CPMV is orally bioavailable when inoculated into mice as infected leaves [9], attempts were also made to inactivate virus within infected leaves prior to purification. Infected leaves showing lesions were irradiated at 7.5 J/ cm^2^, 10.0 J/ cm^2^, or 20.0 J/ cm^2^, and homogenates made from the leaves were suspended in 1.5 ml 0.1 M phosphate buffer and inoculated directly onto primary leaves of five cowpea seedlings per each dosed group. No inhibition of infectivity compared to un-irradiated leaves was observed (data not shown), suggesting that at least at these doses absorbance of the UV irradiation by leaf pigments is sufficient to block the effect of UV on virus infectivity.

### Effect of UV irradiation on viral genomic RNA

**Figure 2:**
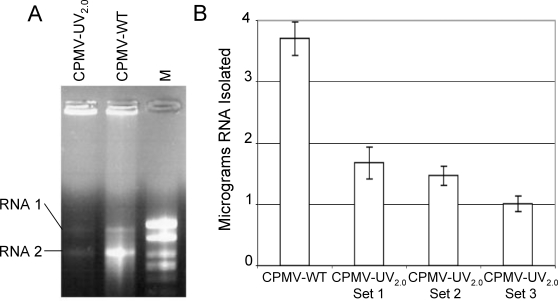
UV-Crosslinking of CPMV genomic RNA. A. Total genomic RNA was isolated using phenol-chloroform extraction from purified CPMV particles either untreated (CPMV-WT) or treated with 2.0 J/cm^2^ of UV radiation (CPMV-UV_2.0_), and equal quantities of purified RNA were separated on an agarose gel matrix. Genomic RNA-1 (5.9 kb) and RNA-2 (3.6 kb) are indicated. M: molecular weight markers. B. RNA was extracted in triplicate from 20 micrograms of CPMV-WT or from three individually UV-inactivated samples of CPMV-UV_2.0_. Results are reported as total viral RNA isolated from phenol-chloroform extractions (mean+/−S.D.). Data are representative of two independent experiments.

To confirm the effect of CPMV inactivation, the integrity of the viral genomic RNA in irradiated samples was investigated. RNA was isolated from CPMV-UV_2.0_ and CPMV-WT whole virus particles by phenol-chloroform extraction and ethanol precipitation, and run on an agarose gel under RNAse-free conditions. RNA-protein crosslinking was observed. When viral protein was extracted from CPMV-UV_2.0_ using phenol-chloroform, RNA yield was reduced, however some viral RNAs remained that migrated with untreated CPMV RNAs (Figure 2A). Nevertheless, quantifying the viral RNA extracted from CPMV-WT and three independently irradiated sets of CPMV-UV_2.0_ resulted in an average 64% lower yield of viral genomic RNA when compared to non-irradiated samples, suggesting that such RNA-protein crosslinking had occurred (Figure 2B). Little is known about the structure of RNAs packaged inside CPMV aside from the fact that each particle can package a single RNA molecule [38]–[40], and in other virus systems RNA-RNA crosslinking has been primarily reported following UV treatment. It is possible inter- or intra- RNA crosslinking may also occur.

### UV-treated particles remain intact following inactivation of infectivity

**Figure 3:**
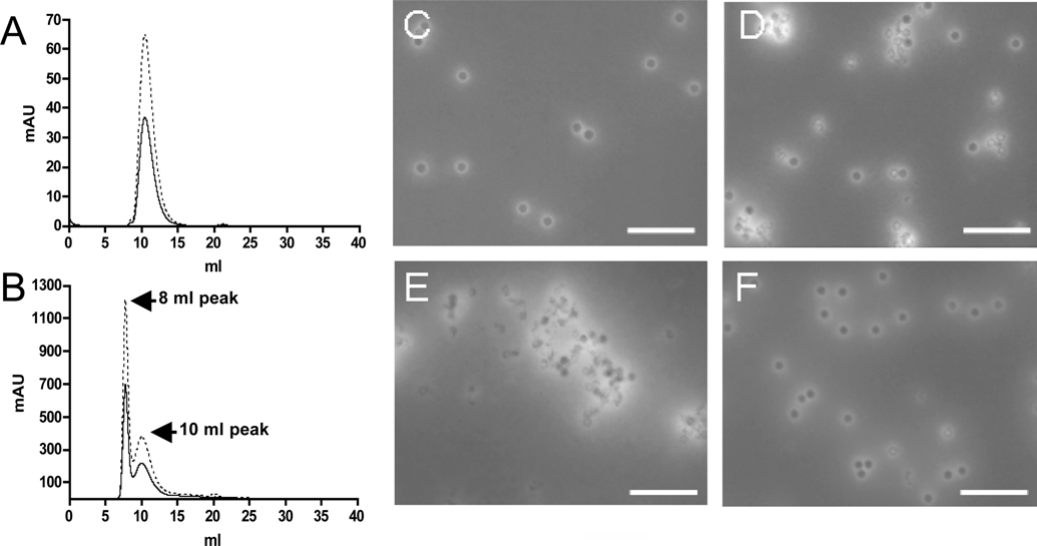
Effect of UV irradiation on capsid morphology by size-exclusion chromatography and TEM. CPMV-WT (A) and CPMV-UV_2.5_ (B) were run on a Sepharose 6 FPLC column. Dotted lines indicate absorbance at 260 nm and solid lines indicate absorbance at 280 nm. Single particles of CPMV-WT typically elute from the column at approximately 10 ml (panel A). CPMV-UV_2.5_ contains aggregated particles that elute earlier in a peak at approximately 8 ml (panel B) as well as intact, non-aggregated particles that elute in a peak at 10 ml. Note the difference in y-axis values reflecting different amounts of particles run on each column. CPMV-WT (panel C) and CPMV-UV_2.5_ (panel D–F) samples were analyzed by TEM. CPMV-UV_2.5_ samples (D) contained more aggregation and debris than CPMV-WT. The CPMV-UV_2.5_ sample was further separated into the 8 ml peak (panel E) and 10 ml peak (panel F) demonstrating the aggregated and single-particle fraction of the UV-treated CPMV. The scale bar represents 200 nm.

CPMV particles that contain wild-type genomic RNAs are highly stable. Empty particles that lack RNA, while they do exist at about 10% of a wild-type infection, are more difficult to produce in large quantities [31]. Artificially-generated top component, produced by high-pH treatment of capsids is relatively unstable, suggesting that CPMV RNA plays a structural role in maintaining capsid integrity [31]. To determine if crosslinking the viral RNAs led to damaged capsid integrity or compromised the particle structure, UV-treated samples were run on a FPLC size exclusion column. Peaks at 260 nm and 280 nm for the CPMV-UV_2.0_ (data not shown) sample produced histograms similar to that of CPMV-WT (Figure 3A), eluting in the same fraction and with an analogous 260∶280 ratio. This observation was further confirmed by SDS electrophoresis where the capsid protein subunits of inactivated and non-inactivated samples were identical (not shown). Likewise, electron microscopy (not shown) demonstrated that UV-treated particles had similar morphology to control samples, and no significant difference in aggregation or in the relative amounts of broken, empty, or partially full capsids was observed.

In contrast, CPMV-UV_2.5_ samples showed increased aggregation, and displayed a separate peak that eluted earlier than wild type by size-exclusion chromatography (Figure 3B). These results suggested that some particle disruption was generated by the higher-dose UV exposure and that these broken particles then could aggregate. The morphology of the total CPMV-UV_2.5_ fraction was then compared to CPMV-WT by TEM (Figure 3, panels C and D). The images showed aggregates and debris in the CPMV-UV_2.5_ fraction (Figure 3D) that was not observed in CPMV-WT (Figure 3C). Further fractionation of the CPMV-UV_2.5_ sample into 8 ml (Panel E) and 10 ml (Panel F) peaks confirmed that the 8 ml fraction contained the aggregated CPMV particles and the 10 ml peak contained the single particles with morphology similar to untreated CPMV-WT. Additional experiments showed that the yield of single, non-aggregated particles could also be improved via the use of 0.22 µm filtration of the particles prior to ultracentrifugation, along with the addition of 5 mM EDTA, however this was not as effective as size-exclusion chromatography (data not shown).

### UV-inactivated particles retain surface chemical reactivity

**Figure 4:**
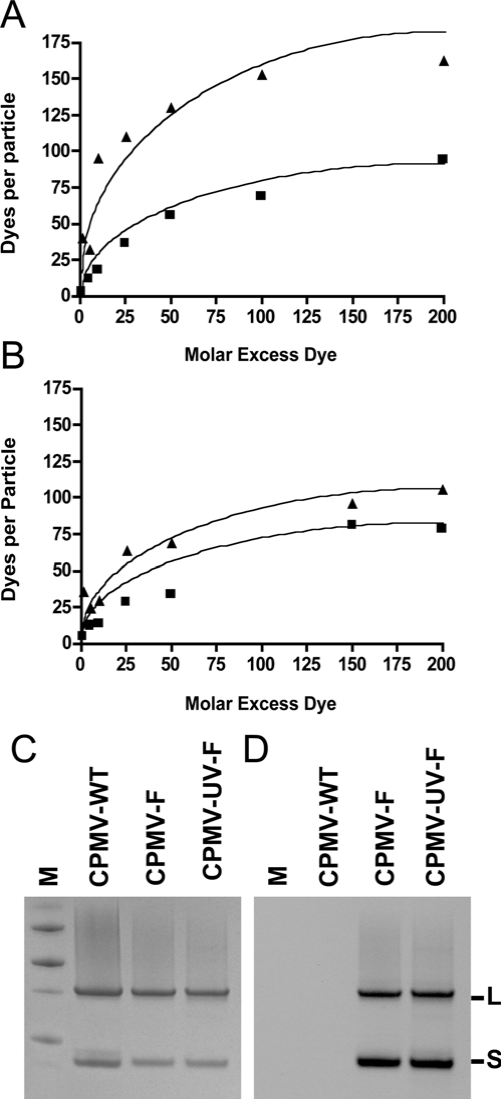
Surface chemical reactivity of CPMV-UV. CPMV-WT (squares) and CPMV-UV_2.5_ (triangles) samples were labeled with varying amounts of excess NHS-Fluorescein (ratio of dye molecules to asymmetric unit; x-axis), and following purification the dyes per particle were calculated (y-axis). Panel A shows labeling of CPMV-UV_2.5_ samples prior to FPLC fractionation. Panel B shows labeling of CPMV-UV_2.5_ where the 10 ml fraction (as in Figure 4) was first purified by FPLC prior to labeling. CPMV-F and CPMV-UV-F that had been labeled with NHS-fluorescein were run on SDS PAGE. Both large and small capsid subunits remain intact (C) and fluoresced under UV light (D).

Having demonstrated that capsid integrity was essentially maintained following UV exposure, we then studied the ability of CPMV-UV_2.5_ particles to react with an amine-reactive fluorescent dye, which conjugates to exposed lysines on each asymmetric unit of the CPMV capsid. Lysine reactivity on the particle exterior surface is essential for a variety of techniques used for CPMV nanoblock chemistry including azide-alkyne [3+2] cycloaddition [28]. CPMV-UV_2.5_ samples were incubated with *N*-hydroxysuccinimide (NHS)-fluorescein at ratios of 1, 5, 10, 25, 50, 100, and 200 dyes molecules/asymmetric unit, in comparison to CPMV-WT labeled in the same manner. Following removal of unconjugated dye, the number of dyes/particle was calculated and compared with CPMV-WT. CPMV-UV_2.5_ samples demonstrated an increase in ability to react with the dye, producing dyes per particle values approximately two-fold greater than the untreated samples (Figure 4A).

Because the CPMV-UV_2.5_ sample contained aggregates as described above, the reactivity of a pure population of UV-inactivated, single particles (10 ml fraction from Figure 4B) was also investigated. The purified CPMV-UV_2.5_ particles were labeled with excess amounts of NHS-fluorescein and the labeled dyes per particle calculated and compared with CPMV-WT labeled in parallel with the same amounts of dye. The purified CPMV-UV_2.5_ samples produced dyes per particle values similar to CPMV-WT (Figure 4B). SDS-PAGE analysis was used to further demonstrate the retention of surface reactivity. Fluorescein-labeled CPMV-UV_2.5_ (CPMV-UV_2.5_ -F), CPMV-WT (CPMV-F) and unlabeled CPMV-WT samples were run on a 12% SDS-PAGE gel and visualized under UV light. Fluorescence was observed in both large and small subunits of CPMV-UV_2.5_ and the bands appeared identical to the CPMV-F sample (Figure 4D). Protein staining also showed indistinguishable bands among control and UV-treated samples (Figure 4C).

### UV inactivation does not reduce the efficiency of cell binding and uptake *in vitro*

**Figure 5:**
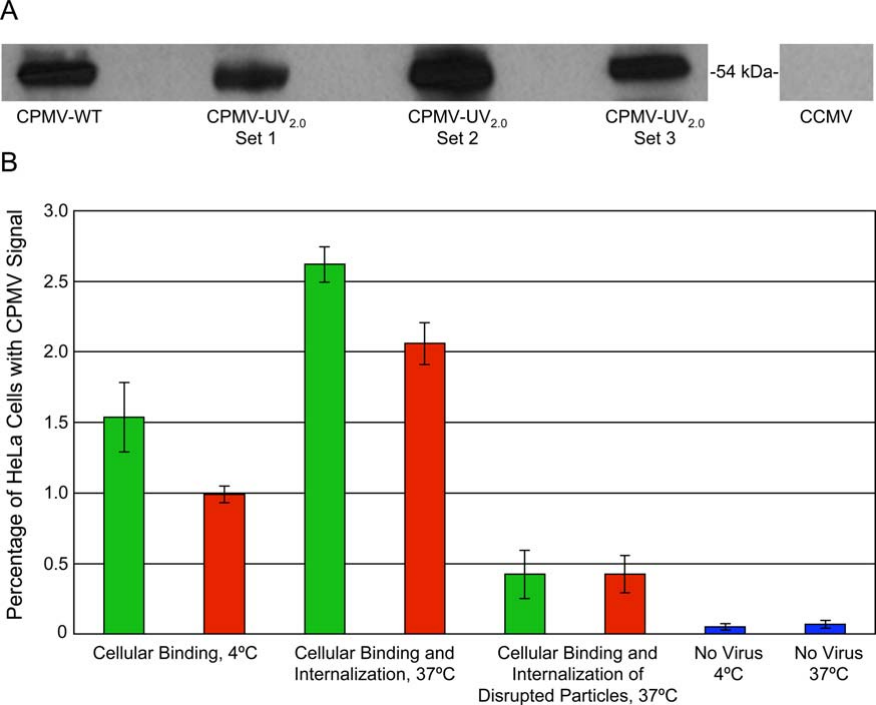
Cell-surface binding and uptake of CPMV-WT and CPMV-UV_2.0_. A. CPMV-WT and the three CPMV-UV_2.0_ samples used in figure 2B were incubated separately with the plasma membrane enriched fraction of HeLa cells and virus binding to an established CPMV binding protein (CPMV-BP) was visualized through VOPBA. No binding was seen when CCMV was used in place of CPMV. B. CPMV-WT (green bars) and CPMV-UV_2.0_ (red bars) particles were incubated with HeLa cells for 2 hours at 4°C to measure cellular binding or at 37°C to measure binding and internalization. Respective disrupted particles were incubated at 37°C as well. Percentage of cells with CPMV signal were detected by flow cytometry are indicated on the y-axis and data are reported as mean+/−S.D. of triplicate samples.

Previous studies indicated that UV irradiation of the picornaviruses poliovirus and hepatitis A virus abolishes their binding to cell surface receptors [41]. For CPMV, studies have demonstrated that wild-type and fluorophore-labeled CPMVs are endocytosed in a variety of cell types both *in vitro* and *in vivo* [9]–[11]. In addition CPMV is known to bind to a 54 kilodalton mammalian surface protein (CPMV-BP) [42]. Using a Virus Overlay Protein Blot Assay (VOPBA), binding of CPMV-UV_2.0_ and CPMV-WT particles to CPMV-BP from HeLa human epithelial cell plasma membranes was tested indicating no difference in binding capacity (Figure 5A). When cowpea chlorotic mottle virus (CCMV), a similarly structured virus that infects the same plant host, was used in place of CPMV no binding in the 54 kilodalton region was observed (Figure 5A) [42]. Triplicate samples of HeLa cells were also incubated with 10^5^ particles of CPMV-WT (green bars) or CPMV- UV_2.0_ (red bars) per cell for two hours at 4°C to illustrate binding or at 37°C to show binding and internalization (Figure 5B). Heat-disrupted capsids showed little binding and uptake when incubated with HeLa cells at 37°C (Figure 5B). UV-inactivated particles are still capable of near wildtype cellular binding and uptake *in vitro*. These data further indicate that unlike other picornaviruses, the CPMV capsid remains stable and intact, and maintains surface reactivities and cellular binding properties following UV irradiation.

## Discussion

In this study, it was shown that CPMV plant infectivity could be inactivated using 254 nm UV irradiation, while preserving the structural integrity and chemical reactivity of the capsid. The viral genomic RNA was extensively crosslinked following UV treatment. Doses that resulted in complete inactivation of infectivity caused some breakdown and aggregation of a portion of the virus population. Removal of these aggregates was required prior to chemical labeling to achieve results similar to untreated CPMV-WT. Other methods that result in inactivation of picornaviruses and other nonenveloped viruses include sodium hypochlorite, formaldehyde, and proteinase K. In our study these methods either did not affect CPMV infectivity, or compromised capsid structure or chemical reactivity (not shown). Together these results indicate that UV is an appropriate method to inactivate CPMV, but that conditions that minimize particle destruction must be fine-tuned in order to maintain particle morphology and reactivity to bioconjugation.

Previous studies using CPMV for vaccine applications showed that immunogenicity of CPMV particles was retained following exposure to 1.8 J/cm^2^ of UV at 1 mg/ml [5]. In that study the morphology of the particles following UV treatment was reported to be intact by SDS-PAGE and EM, and the ability of UV-treated virus to replicate was measured in the reporter host *Chenopodium amaranticolor*. However, no studies investigating the capsid chemical reactivity were conducted. In the present study more UV was required to demonstrate the absence of symptoms in the primary host, *V. unguiculata*, which had to be increased to 2.0 J/cm^2^ in order to prevent systemic spread in plants. Titration of the amount of infectious virus required to observe symptoms *in vivo* showed that an inoculum of 10^8^ (1 ng) CPMV particles is required for observing infection in inoculated *V.unguiculata* leaves. The difference in UV dose required for inactivation could be a feature of differences in UV sources, or differences in the particle concentration during UV treatment. Indeed our studies found that at virus concentrations greater than 2 mg/ml it was increasingly difficult to inactivate infectivity even with the higher doses of UV. These results suggest that the capsids themselves absorb UV and protect the viral RNA. Finally, since the vaccine studies were performed using CPMV chimeras that display heterologous epitopes and the S protein displaying these epitopes is cleaved on the capsid surface, it may be that chimeric CPMV particles are less stable to UV irradiation than CPMV-WT [5]. Thus it is important to standardize the method of UV inactivation of CPMV and CPMV chimeras, taking into account the dose, volume, and concentration of virus when preparing particles for *in vivo* use.

Surprisingly, although CPMV is known to be highly stable, even relatively low doses of UV lead to some particle aggregation and breakage. This is similar to a recent study of picornavirus inactivation by UV, showing that at doses of approximately 1.25 J/cm^2^ poliovirus capsids could not protect the genomic RNA from subsequent nuclease treatment, and the UV-treated particles could not attach to cellular receptors [41]. Our study indicates that further purification of CPMV particles following UV inactivation is required to ensure that intact particles are being administered *in vivo*. Indeed, in samples where aggregated and broken particles are removed by size-exclusion chromatography, the chemical reactivity of surface lysines is only slightly higher than untreated particles. These results also suggest that perhaps internal or inter-subunit lysine residues, which are normally not exposed in intact particles, may be exposed following UV treatment as a result of RNA crosslinking and subtle changes in capsid morphology.

The further development of CPMV for *in vivo* applications such as nanoparticle-based imaging sensors, therapeutics, and vaccines depends on the ability to effectively standardize the properties of CPMV preparations. The results presented here indicate that efficient inactivation of CPMV particles is feasible for *in vitro* and *in vivo* use. Nevertheless it is clear that precise conditions for inactivation must be determined for maintaining particle structure and chemical reactivity.

## Materials and Methods

### Preparation and inactivation of purified cowpea mosaic virus (CPMV)

Virus was purified from wildtype-infected leaves of Kentucky cowpea (Vigna unguiculata) plants by a method previously described [9]. Purified virus particles were resuspended at 2 mg/ml in 0.1 M phosphate buffer (pH 7.0) containing 5 mM EDTA. Aliquots were irradiated in a Stratalinker 1800 UV Crosslinker (Stratagene, La Jolla, CA) equipped with 254 nm UV bulbs (5 at 8 watts each) delivering 3000 µwatts/cm^2^. Samples were placed in 100 cm open petri dishes 15 cm from the light sources, irradiated in energy mode at doses of 0.06 J/cm^2^, 0.12 J/cm^2^, 0.18 J/cm^2^, 0.36 J/cm^2^, 0.72 J/cm^2^, 1.00 J/cm^2^, 2.00 J/cm^2^, or 2.50 J/cm^2^, and subsequently stored at 4°C for later use. Disruption of CPMV-UV_2.0_ and CPMV-WT particles was completed by incubation at 95°C for 30 minutes.

### Infectivity of irradiated CPMV on *V. unguiculata* or *Phaseolis vulgaris* var. pinto seedlings

Individual samples which had received varying doses of UV were used to infect cowpea seedlings where n = 6/sample. The primary leaves of nine-day-old cowpea plants were bruised with carborundum and infected with either 2.5 µg inactivated or non-inactivated control CPMV particles per plant at a concentration of 25 ng/µl in 0.1 M phosphate buffer. Inactivated samples were later used to infect leaves with 5.0 µg per plant at a concentration of 50 ng/µl. Infections were monitored for the appearance of lesions and diminished growth in secondary leaves for 16 days post-inoculation. For infections on pinto bean seedlings, primary leaves were inoculated with 100, 10, or 1 ug of CPMV that were non-irradiated or irradiated at 2.0 J/cm^2^. Symptoms were observed at 1 week post-inoculation and lesions quantitated on inoculated leaves using an AlphaInnotech imaging system.

### FPLC analysis and fractionation of wild-type and UV-inactivated CPMV

Samples were analyzed by FPLC using an AKTA Explorer Superose™ 6 size-exclusion column (Amersham Pharmacia) in which particles suspended in 0.1 M phosphate buffer (pH 7.0) were run at a rate of 0.4 ml/min and absorbance was measured at 260 and 280 nm. For preparative size-exclusion FPLC, intact particles were separated from aggregated and broken CPMV particles through collection of fractions that coincided with the 10 ml elution peak of untreated CPMV particles. These fractions were collected, pooled and concentrated by ultracentrifugation at 42,000 rpm in a 50.2Ti rotor for 3 hours. Results presented are representative of more than 6 independent experiments.

### Comparison of chemical addressability of CPMV-WT and CPMV-UV

CPMV-UV_2.5_ was purified by FPLC fractionation and then labeled with *N*-hydroxysuccinimide (NHS)-Fluorescein, [5-(and 6)-carboxyfluorescein succinimidyl ester] (Pierce Biotechnology Inc, Rockport, IL) in parallel with CPMV-WT. 200 µg of virus at a concentration of 1 mg/ml in 10% DMSO was mixed with various amounts of excess NHS-Fluorescein relative to viral asymmetric subunits (60/particle) in the following ratios: 1, 5, 10, 25, 50, 100, 150, and 200. The reaction was carried out in a volume of 200 µl with gentle agitation for 24 hours at room temperature. Unbound dye was separated from labeled particles using a 10%– 40%sucrose gradient in an SW41 rotor for 2 hrs at 40,000 rpm (Beckman). Bands of intact, labeled virus were recovered from the gradient and concentrated by ultracentrifugation in a Ti50 rotor for 3 hrs at 42,000 rpm. Samples were resuspended in 0.1 M phosphate buffer and absorbance measured at 260, 280 and 494 nm using a DU 800 spectrophotometer (Beckman Coulter). Dyes per particle was calculated with the following equation: dyes/particle = [(Abs_494_×dilution) / ∈_Fluorescein_] / (CPMV concentration / CPMV molecular weight); where ∈_Fluorescein_ = 74,200 and the molecular weight of CPMV = 5.6×10^6^ grams/mole.

To visualize labeled CPMV proteins, 6 µg samples of CPMV-UV_2.5_ and CPMV-WT were mixed with 5 µl 4× LDS with DTT sample buffer (NuPAGE, Invitrogen) and run on a NuPAGE 12% Bis-Tris Gel (Invitrogen). The gel was visualized under UV light in a FluorChem SP imaging system (Alpha Innotech) to detect the fluorescence of the labeled samples, followed by protein detection using Simply Blue Safe Stain (Invitrogen).

### Transmission electron microscopy

CPMV-UV_2.5_ and CPMV-WT samples were analyzed. The CPMV-UV_2.5_ samples were further fractionated by Sepharose 6 FPLC and the fractions corresponding to aggregates (8 ml elution volume) and single particles (10 ml elution volume) were also visualized separately. Carbon-coated, copper grids were glow discharged immediately before loading samples. Grids were placed in 10 µl of virus sample for 1 minute, washed three times in sterile filtered water, and stained in 10 µl drops of 1% uranyl acetate three times, allowing the grids to soak in the last drop of stain for 2 min. After drying grids, samples were examined under an electron microscope (Phillips CM100) at 52K magnification.

### Analysis of CPMV genomic RNA following UV treatment

Genomic RNA was isolated from CPMV-UV_2.0_ and CPMV-WT particles by standard phenol-chloroform extraction of virus particles followed by ethanol precipitation under RNAse free conditions. Viral genomic RNAs were analyzed on a 1% agarose RNAse-free gels (Apex) under native conditions, and visualized under UV light in a FluorChem SP imaging system (Alpha Innotech). RNA extractions were performed in triplicate with mean+/−S.D. reported.

### Flow cytometry

Intact and disrupted CPMV-UV_2.0_ and CPMV-WT particles were incubated with HeLa human epithelial cells cultured in Dulbecco's minimal essential media (DMEM) supplemented with 10% fetal bovine serum (FBS), 1% L-glutamine, and 1% penicillin/streptomycin. Samples were incubated in parallel at a concentration of 10^5^ viruses per cell for a period of 2 hours at 4°C or 37°C. Samples were then fixed with 2% paraformaldehyde, and CPMV identified through fluorescent antibody detection and analyzed by flow cytometry. Cells were acquired on a LSRII flow cytometer (10,000 events) and analyzed with FlowJo software (Treestar, San Carlos, CA) in triplicate. Error bars indicate mean+/−standard deviation (Microsoft Excel).

### VOPBA

HeLa cells were propagated and plasma membranes isolated and stored in 10 mM Tris/HCl, pH 8.0, 10 µg/mL aprotinin and leupeptin (Roche), and 0.5% n-octyl-β-D-glycopyranoside (Sigma) as described previously [42]. 10 µg of plasma membrane protein isolates were run on 4–12% Bis-Tris 1.0 mm NuPAGE gel (Invitrogen). Proteins samples were then transferred electrophoretically to Immobilon-P transfer membranes (Millipore). Transfer membranes were then blocked overnight with 5% w/v milk solution diluted in wash buffer consisting of PBS with 0.2% Triton X-100 (Sigma). Samples were then separately subject to one hour incubation with 10 µg/mL of CPMV-UV_2.0_ or CPMV-WT in 1% milk solution with 5% glycerol, washed 4 times with wash buffer for 5 minutes each, then subject to one hour incubation with anti-CPMV polyclonal antibody, washed 4 times with wash buffer for 5 minutes each, then incubated one hour with goat anti-rabbit IgG-HRP, washed 4 times with wash buffer for 5 minutes each, visualized with chemiluminescence detection (SuperSignal; Pierce) and exposed on a single CL-Xpossure film (Pierce). The cowpea chlorotic mottle virus (CCMV) VOPBA was conducted using the same method with appropriate primary and secondary antibodies for CCMV detection. Both purified CCMV and the polyclonal rabbit anti-CCMV antibody were generous gifts from Mark Young.

## Supporting Information

Figure S1 Infectivity of UV-inactivated CPMV in a local lesion host Phaseolis vulgaris var. Pinto (pinto bean). For infections on pinto bean seedlings, primary leaves were inoculated with 100, 10, or 1 ug of CPMV that were non-irradiated or irradiated at 2.0 J/cm2. Symptoms were observed at 1 week post-inoculation and lesions quantitated on inoculated leaves using an AlphaInnotech imaging system. Primary leaves (panels A–C and E–G) were inoculated with CPMV and the presence of symptoms were monitored. Leaves were inoculated with CPMV with the following doses of UV irradiation: 0 J/cm2 (positive control; panels A–D), 2.0 J/cm2 (panels E–H). Lesions per inoculated leaf were quantitated in panels D and H. Bars represent mean+/−S.D. of 4 leaves/sample.
(1.30 MB TIF)

Text S1 (0.65 MB DOC)
