# Supplementary figures and images for: Chemical Addressability of Ultraviolet-Inactivated Viral Nanoparticles (VNPs)
Source: PLoS One. 2008 Oct 2;3(10):e3315. doi: 10.1371/journal.pone.0003315 (PMC2551747; doi:10.1371/journal.pone.0003315)

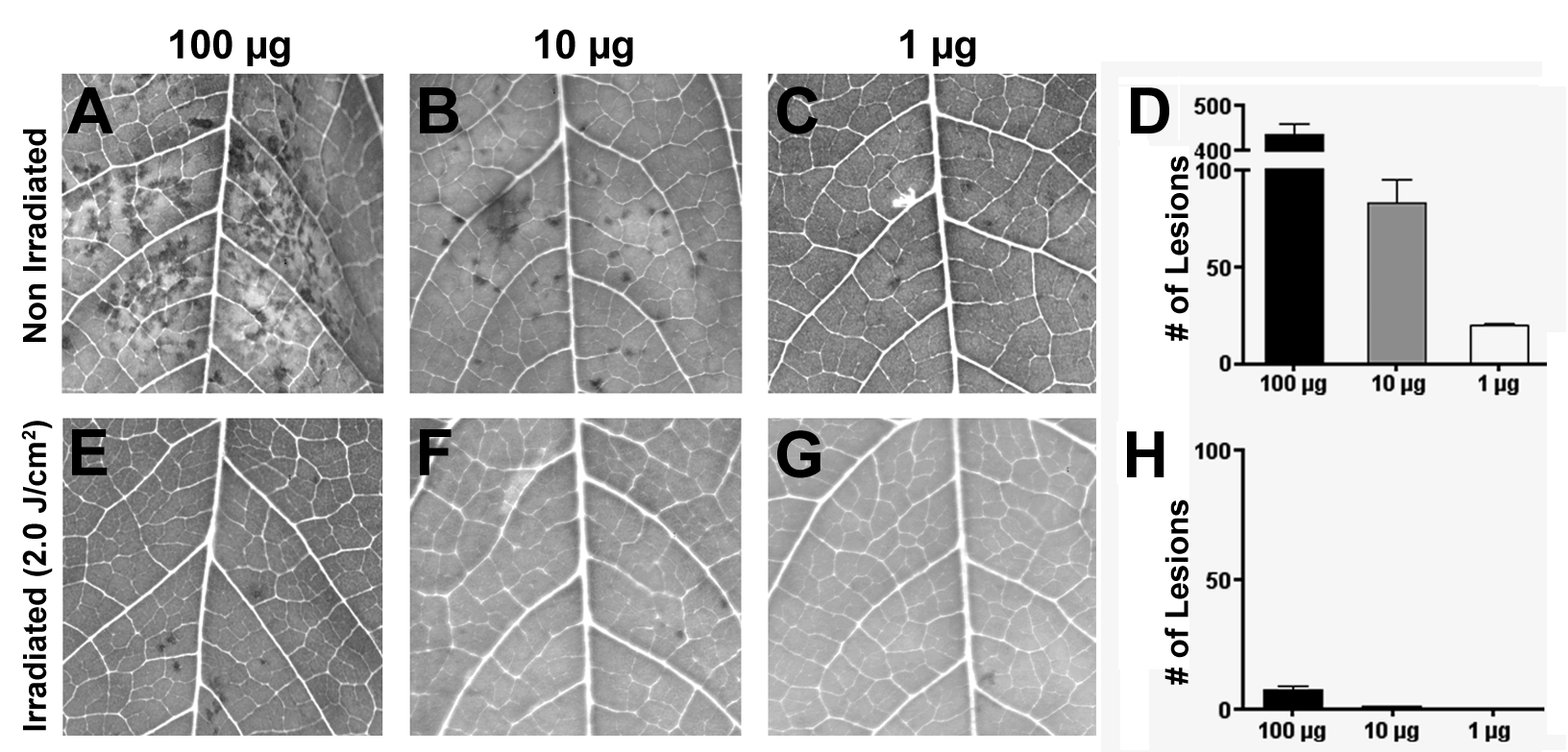

Supplement: Figure S1 — Infectivity of UV-inactivated CPMV in a local lesion host Phaseolis vulgaris var. Pinto (pinto bean). For infections on pinto bean seedlings, primary leaves were inoculated with 100, 10, or 1 ug of CPMV that were non-irradiated or irradiated at 2.0 J/cm2. Symptoms were observed at 1 week post-inoculation and lesions quantitated on inoculated leaves using an AlphaInnotech imaging system. Primary leaves (panels A–C and E–G) were inoculated with CPMV and the presence of symptoms were monitored. Leaves were inoculated with CPMV with the following doses of UV irradiation: 0 J/cm2 (positive control; panels A–D), 2.0 J/cm2 (panels E–H). Lesions per inoculated leaf were quantitated in panels D and H. Bars represent mean+/−S.D. of 4 leaves/sample. (1.30 MB TIF) [file pone.0003315.s001.tif]
