# Supplementary material for: Chemical Addressability of Ultraviolet-Inactivated Viral Nanoparticles (VNPs)
Source: PLoS One. 2008 Oct 2;3(10):e3315. doi: 10.1371/journal.pone.0003315 (PMC2551747; doi:10.1371/journal.pone.0003315)
Supplement: Text S1 — (0.65 MB DOC) [file pone.0003315.s002.doc]

**Supporting information.**

**Infectivity of UV-inactivated CPMV in a local lesion host.**

Additional experiments comparing infectivity of CPMV were performed using a local lesion host for CPMV, *Phaseolis vulgaris var. pinto* (pinto bean), a host that permits CPMV replication in inoculated primary leaves but does not permit systemic spread of virus. Non-irradiated CPMV showed dose-dependent symptoms on inoculated leaves (Figure S1), whereas CPMV irradiated with 2.0 J/cm^2^ showed an approximately 50-fold decrease in symptoms in inoculated leaves (Figure S1).
